# Community perception of the determinants of unmet needs of family planning among married women in Buea Health District, Southwest Region, Cameroon

**DOI:** 10.11604/pamj.2023.45.58.33949

**Published:** 2023-05-25

**Authors:** Layu Donatus, Tendongfor Nicholas, Dohbit Sama Julius, Egbe Thomas Obinchemti

**Affiliations:** 1 Department of Public Health and Hygiene, Faculty of Health Sciences, University of Buea, Buea, Cameroon,; 2 Department of Obstetrics and Gynecology, Faculty of Medicine, University of Yaoundé, Yaoundé, Cameroon

**Keywords:** Community perception, unmet need, contraception, Buea Health District

## Abstract

**Introduction:**

in Cameroon, and more specifically in the Buea Health District of the Southwest Region, there are still a few unmet family planning needs. Many women desire to avoid getting pregnant, but do not use an effective form of birth control. A focus group discussion among married women in the Buea health district was necessary to explore the determinants of unmet family planning in order to promote access to and use of long-acting modern contraceptive methods because most research studies have only focused on the quantitative aspect.

**Methods:**

focus groups were held in the community and in the medical facilities as part of the study's qualitative exploratory strategy. To invite the participants, invitations were sent out. A qualitative survey of 10-12 respondents was carried out by the researcher, in each Focus Group the discussed topics included factors that encourage the use of FP methods, making the switch from traditional to modern FP methods, family planning methods decision-making, accessibility of FP in the context of the COVID-19 pandemic. Each focus group discussion lasted 1 hour 30 minutes, the perception was collected in an audiotape recorder and later transcript verbatim. The team conducted 10 Focus Group Discussion (FGD) (four of each of the topic areas). The FGD team provided participants with light refreshments.

**Results:**

a total of 10 focus groups were conducted, reaching a total participant of 107. The N-Vivo analysis software was used to analyze the data. The following are some of the key participant perceptions that have been reported. Perceptions of family planning; for health reasons as well as social expectations and pressures, having children earlier in marriage was a wise decision. While there are certain issues with having children early in life, such as the mother's maturity and financial stability, having a kid as soon as feasible is the best option. It is best to have children within the first two years of marriage, with a three-to-five-year gap between them. Reasons for changing from traditional to modern family planning; The most significant issues with current birth control techniques (such as condoms, pills (postinor-2), implants, injectables, and IUDs) are those that affect a woman's body. Regardless of the issues, they experience with contraception, there are some benefits to utilizing it. Both modern and classic FP methods have the potential to fail. Making family planning decisions; extended families, particularly mothers-in-law, have a strong influence on family planning and size decisions, and family members assist in resolving FP issues and challenges. Participants want to know about the adverse effects of current birth control techniques, especially in the long run, and how contraceptives affect a future pregnancy. Accessibility of FP in the context of the COVID-19 pandemic; participants have mixed perceptions of whether the COVID-19 context influences their accessibility to family planning.

**Conclusion:**

the focus groups demonstrated that participants have a nuanced and sophisticated awareness of pertinent topics that significantly impact them. While cultural and social conventions surrounding family planning difficulties and decision-making continue to put pressure on women, traditional and modern family planning methods are well-known. Participants also reported a strong desire to learn more about family planning alternatives, the effects of different methods on their bodies, and to have access to a wider selection of general and specialized family planning services. Fear was produced by the backdrop of COVID-19, but women are rapidly overcoming these fears to have access to family planning.

## Introduction

Among the 1.9 billion women who will be of reproductive age (15 to 49) around the world in 2020, many women and couples wish to delay or avoid getting pregnant [1]. In order to limit or delay having children, 1.1 billion women are regarded to need family planning, and 851 million of these women use a modern method of contraception while 85 million use a traditional technique [1]. Despite wanting to avoid getting pregnant, an additional 172 million women use no technique at all and are therefore thought to have unmet family planning needs [1,2]. Despite declines in the global percentage of women with unmet needs for family planning, the global number of women with unmet needs for family planning has increased due to the continued growth in the size of the population of women of reproductive age [2]. In sub-Saharan Africa, future population growth will pose challenges to expanding coverage of reproductive healthcare services from 2020 to 2030, sub-Saharan Africa will see the largest increase (60 percent) in the number of users of modern contraceptive methods, driven both by the continued increase in the absolute number of women of reproductive age and by the increase in contraceptive prevalence [3]. In the Central African region, the rate of use of modern contraceptive methods is 18% among married women and the unmet need for modern contraceptive methods is over 24% [3]. Sixty-three percent of women in Cameroon who want to prevent pregnancy do not utilize a modern form of contraception [4]. Most of the studies in Cameroon focus on rural and urban women at the individual and household levels, hence the community perception is often neglected. In 2013, an estimated 40% of pregnancies in Cameroon were unintended. More than six in 10 women who want to avoid pregnancy either do not practice contraception or use a relatively ineffective traditional method [4]. If all unmet needs for modern methods were satisfied, maternal mortality would drop by more than one-fifth, and unintended births and unsafe abortions would decline by 75% [4-6]. Therefore, the goal is to identify the factors that contribute to unmet needs for contemporary family planning use among Buea Heath District reproductive-age women (15 to 49 years) old who are married or in a consensual relationship). This discovery will support the use of and access to long-acting modern contraceptives.

## Methods

**Figure 1:**
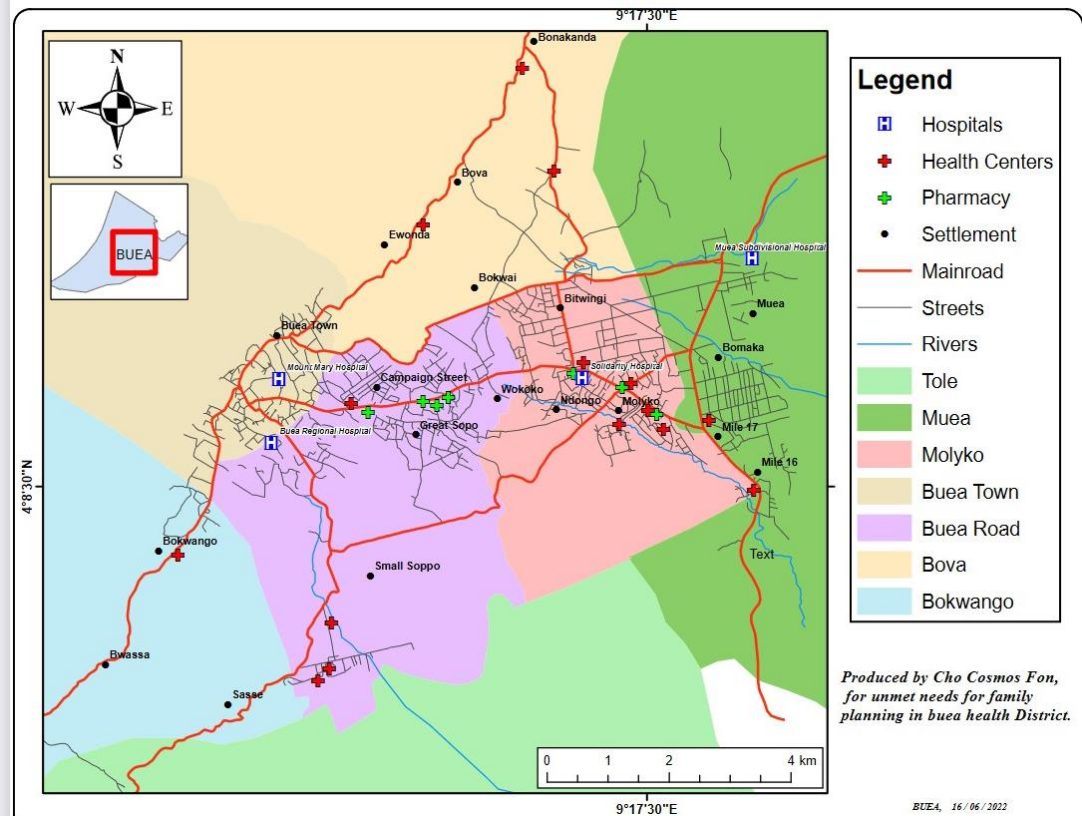
map of Buea Health District

**Study setting:** the study was conducted in the seven health areas of the Buea Health District (BHD), situated in the Fake division of the South West Region of Cameroon (Figure 1). Buea is the administrative capital of the South West Region of Cameroon. The town is located at the base of Mount Cameroon and has 85 villages (including the villages of Bokwaongo, Muea; Bomaka; Tole; Mile 16 (Bolifamba); Mile 17; Mile 15; Mile 14 (Dibanda); Bova; Bonjongo; Likombe; Buasa; Great Soppo; Molyko; Small Soppo; Bwitingi; Mile 18 (Wonyamavio); Lower farms; Bokwai; Bonduma; Sandpit, Wonyamongo, Bulu; Bokova and surrounding villages). It has a Regional Hospital (secondary level) which serves as the region´s referral hospital, seven primary care facilities, and a few private hospitals. The study setting was BHD in selected health facilities and some homes in the community. In the hospitals, other health workers also participated and in the community, the head of the homes where the FDGs sessions were held participated. The sample consisted of women 15 to 49 years, who are married or in a consensual relationship and resided in the health area of the study at the time the study was carried out. A pilot test of the FGDs question guide was carried out and corrections were done. The questions were prompt and stimulating to get the best response from the participants. Reported were delegated, and field notes were taken, the audio recorder was also used. Each discussion had questions asked and each time the group unanimously agreed with the response it was noted as the final response. Saturation was reached as questions were repeated for 4 times, after which the final reposed was read and corrections done. The focus group discussions lasted one and a half hours.

**The research team and flexibility:** the focus ground discussion was conducted by a team of 8 researchers, the team consists of 01 professor, 2 Ph.D. Students, 3 MPH students, and 2 sociologist Ph.D. Student. At the time of this research, the profession was the supervisor and the rest of the research team were Ph.D. students in public Health. The team was made up of 6 males and 2 females. The research team had a two-day orientation on the FGDs questions, and they received training on family planning and the FDGs questions were reviewed. The researchers could speak English and pidgin English and some native languages and know about the community and the health facilities, which enable the researchers to connect to the study participants. This training was done at Malingo Tab (Nso Meeting Hall), and 7 data collectors were trained by the researcher for two days. The method used the adult learning theory (andragogy) for easy apprehension and comprehension of the study rationale and the language of training was English, however, Pidgin English was also used to clarify certain concepts on local language.

### Study design

**Methodological orientation and theory:** the methodological orientation for the study was phenomenological, which was focused on discussing the phenomenon of non-use of modern family planning, which intends to lead to the unmet needs of family planning in the community. Invitations to participate in FGDs were sent out to selected participants two weeks before the chosen date. The sessions were conducted in a conference room in the hospital and some selected houses within the community/quarter where participants resided. Focus Group Discussions (FGD) had seven members, including the researcher as the moderator, a recorder/assistant moderator, and five members of the concerned issue. FGDs lasted a maximum of one and a half hours. The purpose of the Buea Health District's determinants of unmet family planning needs study was to promote access to and use of long-acting contemporary contraceptive methods. The focus groups focused on four topics: (a) factors that influence FP use, (b) traditional vs. current methods, and (c) the FP decision-making process. (d) COVID-19 impact on family planning use. The research team consisted of public health professionals and a sociologist who conducted the focus groups. The project team created three discussion guidelines for focus groups.

**Participants selection:** a purposive sampling technique was used to select participants. A focus group guide was adapted from the USAID Reports in Pakistan [5]. This guide captured data on variables such as sources of information, general family planning information, family planning perceptions, the motive for switching to contemporary family planning from traditional methods, family planning choices, subsequent sexual behavior, risk-taking behaviors, and use of sexual health services. Ten focus group discussions included 10 to 12 participants each for the homogenous couples. The study selected participants using the purposive and the venue for the FDGs was based on how convenient participants were and the participants who gave their consent (Table 1). The participant was informed two weeks giving the date, time, and venue (home or hospital) where the FDG sessions will take place. Each session consisted of 10 to 12 participants. A total of 10 FGDs were conducted, reaching a total of 107 participants as the sample. They were no drop out from the focus group discussion due to the fact that after the sessions participants were given snacks and transport to a ceiling amount of 1000frs. However, other community women who were not invited also came for the FGDs and could not be excluded. The teenage mothers refused to attend because of stigmatization and fear of being criticized by other women.

**Table 1:** profile of participants in FDG at hospitals and community

Topic area	Location		Number of participants	Number of FGD	Number of children	Average age participants	Numbers
Motivators for using FP methods	CMA Muea	Hospital	11	2	1-6	40	#1
			10		1-3	31	#2
		Community	10	2	1-4	30	#3
			12		2-4	27	#4
Switching from traditional to modern FP methods	CMA Molino	Hospital	10	2	1-5	37	#5
			12		1-5	28	#6
		Community	11	2	1-4	31	#7
			10		1-4	32	#8
Decision-making for FP methods	CMA Buea town	Hospital	11	2	1-5	34	#9
		Community	10		2-5	30	#10
Total FGD			107	10			

**Targeted population:** participants of the study were fecund sexually active women married or in a consensual relationship aged 15 to 49 years of age who attended health facilities and in the community. Consent was sorted and those who accepted were considered in BHD to better understand the factors that influence unmet modern family planning needs.

**Inclusion criteria:** a participant of this study was a member of the health facilities and community selected for the study and who accepted to participate in the study after informed consent. All the participants selected for the study participated in the FDG.

**Exclusion criteria:** individuals who refused to participate in the study after the consent, infecund, and whose pregnancies were planned were excluded.

**Development of data collection tool:** a focus group guide will be adapted from the USAID Reports in Pakistan [7]. This guide captures data on variables such as sources of information, general family planning information, family planning perceptions, motivation to switch from traditional to modern family planning, family planning decision-making, subsequent sexual behavior, risk-taking behaviors, and use of sexual health services. 10 focus group discussions were carried out. These will include 10 to 12 each for the homogenous couples.

**Ethical clearance:** the Institutional Review Board of the University of Buea's Faculty of Health Sciences granted ethical approval (2021/1524-10/UB/SG/IRB/FHS). The study protocol will be thoroughly described to the participants. They will be informed about the consequences and rewards of voluntary engagement. This study will take confidentiality into account. There shall be no discrimination based on any other factor, such as religion. Participants will be able to opt out of the study at any time. Participants will be briefed about the study and given informed permission.

**Administrative clearance:** administrative clearance was obtained from the Southwest Regional Delegation of Health, Buea Health District Services, and the village heads/influential leaders/quarter head. (MINSANTE/SWR/RDPH/PS/520/726.

**Data collection procedures:** invitations to participate in FGDs were sent out to selected participants two weeks before the chosen date. The sessions were carried out in a conference room in the hospital and some selected houses within the community/quarter where participants resided. FGDs will have 7 members including the researcher as the moderator, a recorder/assistant moderator, and 5 members of the concerned issue. Focus Group Discussions (FGDs) will last a maximum of one and a half hours. The purpose of the Buea Health District's determinants of unmet family planning needs study was to promote access to and use of long-acting contemporary contraceptive methods. The results often focus group discussions (FGDs) held by the project to uncover in-depth psychological, sociological, and cultural aspects influencing the adoption of modern family planning (FP) methods among Buea married women of reproductive age are presented in this study.

**Data analysis:** the focus group was thematically analyzed using the qualitative data analysis software N-Vivo; the data were analyzed in N-vivo using the following steps; Review your research questions and/or research approach, read a few transcripts, and write summary memos, create a research journal and develop a coding strategy and Code for the broad topic areas/themes. The themes were identified in advance to the focus group, and the audio recorded was transcript verbatim.

## Results

**Motivators for using FP methods:** the advantages of starting a family young are numerous. Participants in the FGD thought it was morally correct to have children earlier in life. According to one participant: *“Women who have children early in life are often healthier and more able to carry a pregnancy to term without experiencing fatigue, sickness, or other concerns related to their health and which permits the woman to better follow their career and become productive.” #1* Maternal and child health and well-being are implicated. As related by one woman: *“The possibility of getting kids late could result in surgery. If a mother is above 40, the likelihood of having “precious” children rises, and as a result, the percentage of Down syndrome kids significantly rises.” #2* Similar to the last participant, this one thought that the mother's age enhanced the likelihood of congenital abnormalities among infants born to older women. For some women, early pregnancy was encouraged by societal pressures and expectations. Two participants verified this, saying that women must: *“Prove fertility, saying I need to know if I am a woman and know also that I don´t have any complication in my system,” #3* and *"Family values of being blessed and fruitful, as well as keeping the husband and mother-in-law content by having a baby earlier" #1* Another participant said that having children earlier in life would: *"Increase her standing and respect within the community and give her the energy to see them grow and also teach so that at old age they will be of help to me and society." #3*

Several participants thought it to be: *“early childbirth is regarded as the “right thing to do.” #2*. Additionally, many think that getting pregnant is simpler when you're younger. According to one participant: *“younger women are typically more fertile, so they can become pregnant again fast without having to worry about their fertility or the potential effects on their bodies.” #4*. Additionally, it is believed that the mother's body will adjust to pregnancy and recuperate from it more quickly than it will when she is older. The perception of greater interactions with children and other family members is another factor in selecting early pregnancies. According to one participant: *“younger women have greater energy, allowing them to better care for their babies, spouses, and families while also working to support their family…” #5*. Another participant repeated this and related: *“Younger women can teach their children and support them in their education, carry out follows of their performance and guides children properly.” #3*. Further: *“Younger mothers are more able to relate to their children's wants and worries since they are closer in age to them…”*.

**Concerns with having children early in life:** participants in the FGD expressed a range of worries about having kids young. Having children before turning 18 was viewed negatively by certain people. All FGD participants, however, agreed that social pressures and traditions, as well as the desire to have a child as soon as feasible after marriage, outweighed any age-specific concerns. The mother's maturity was the subject of yet another set of worries. As one participant said: *“Very young moms lack the maturity to properly care for their children, and they frequently overlook the child vaccination schedule...” #6* Other participants agreed, saying that; *“Young moms might not be capable of parenting or raising a child and might leave their kids with their own mothers or mothers-in-law.” #4* Also, *“A young mother could lack the patience to handle her child's wants and requests, and she might get bored or angry with them, which could result in child molestation and rage.” #3* Another set of worries centered on the mother's capacity to effectively care for their children. As an illustration, one participant said: *“I worry that if I have too many kids too young, I won't have enough money to put them through school, take care of their health, and raise them properly.” #2* A different participant said: *“Young women frequently work, which makes it difficult for them to have enough energy for both employment and caring for young children...” #1* Other participants made the argument that having children earlier in life limits a mother's mobility and may hinder her from going out, completing her degree, or finding employment.

**Timing of children in marriage:** all FDG participants thought it was best to start a family during the first two years of marriage. This results from perceptions of health problems and social expectations or demands. As one woman exclaimed with pride: *“Within the first five days of her marriage, my buddy became pregnant, which was wonderful for her. It implied that after having her child, her family ceased pressuring her. It indicated she was extremely fertile as well…” #7* Another woman related: *“Since I was the first person in my family to get married, I felt under pressure to give my parents a grandchild as soon as possible-within the first year.” #6* Even the person who thought it would be better to put off having children said: *“Despite wanting to wait a little bit longer, I did manage to get pregnant during the first year of our marriage. There was just so much pressure on me from my neighborhood and family.” #8* However, some participants in each FGD had different opinions. In summary, these women believed that it was preferable to wait to have a more solid bond with the spouse and extended family as well as to have an early period of married life free of the stress of raising children. They said that by doing this, the likelihood of a happy marriage once the kids were born would be increased. Participants claimed that by delaying motherhood, the women had the opportunity to: *“Gives you the time and freedom to live their life.” #1*

**Choices regarding birth spacing:** participants in the FGD agreed that a two- to three-year gap between children is ideal. As might be expected, some participants thought that a shorter period of time (one and a half years or less) was preferable, while others thought that a longer period of time (more than five years) was optimal.

**Various techniques for birth spacing:** participants were asked to list popular birth spacing techniques. Among the responses were: Breastfeeding, Condoms, Implants, Injectables, Emergency Pills, Withdrawal (Coitus Interruptus), and Intrauterine Contraceptive Devices (IUCD) It's significant to notice that neither implants nor injectables were discussed (LAM).

**Planning for birth spacing can be challenging:** participants in the FGD identified social pressure and problems with contraceptives as the two most significant obstacles to spacing pregnancies. The biggest issue that the FGD participant was worried about was contraceptive issues. Injectables, implants, and *“some participants were using an IUD and started to have pain in my back and my uterus. I went to see my doctor and found out I was pregnant. I was so upset. The IUD had not worked, and I was faced with having to give birth again so soon after my first pregnancy.” #8* Other contraception methods have caused difficulties for participants. For instance, one woman noted that she had been using the rhythm method and fell pregnant, so she decided to try the pill. Another woman mentioned the difficulties she had using the IUD, including mid-cycle bleeding. Another woman stated: *“I didn´t like the pill because it caused me stress and tension, but I stayed on it. Then I fell pregnant on it, so after I had the baby, I changed to an implant. The first one I didn´t like, so I changed, and I like the Jadelle I have now.” #9* Although social pressures mentioned by participants were a factor, problems with contraceptives themselves were often considered to be more important. Social pressures took many different shapes. As an illustration, one participant said: *“Particularly female condoms, condoms are not regarded as culturally appropriate methods of contraception...” #6* and *“Generally, husbands are unwilling to use them.” #3* Another participant claimed that even though she had only one child and it had been almost three years after the first one was born, her mother-in-law was pressuring her to have more kids. Her mother-in-law believed that her child needed a sibling to play with.

**Obstacles to reducing the number of births:** participants in the FGD did not generally identify any significant challenges in trying to reduce births. However, participants in all of the focus groups mentioned pressure from extended family members (such as mothers-in-law or grandmothers) and pressure to have a male kid as being pertinent.

### Reasons for changing from conventional to modern family planning methods

**Problems with contemporary birth spacing techniques:** participants in the FGD identified the effects on the woman's body of current birth control techniques (such as the pill, implants, COC, injectables, and IUDs) as the most significant challenges. Participants made comments, which included *“IUD movement or slippage might result in an infection that can impair fertility,” #5* and, *“Thyroid issues can result from the birth control pill.,” #3* and *“Injections can cause hormones to unbalance and cysts in the breast.” #1* There were further participant accounts of: *“One woman I know took the pill for ten days before her body exploded. She was in a great deal of discomfort, and it was filled with water. She thought she was going insane. When she visited the physicians, they immediately removed her from it. She responded poorly to the hormones,” #9* and *“the pills raise blood pressure, produce stress, and tension.” #2* Also *Many of my acquaintances have tried using emergency contraceptives without success. #2* The attendees also discussed issues with IUDs and injectables. One woman, for instance, used an IUD and got pregnant with both her first and second children. She went to see her doctor to get the IUD removed because it was also giving her some pain.

**Benefits of using modern birth spacing methods:** despite whatever issues women may have with contraceptives, FGD participants reported that there are still a number of advantages to utilizing them. These benefits include: being able to relax and space pregnancies (more control); giving the woman time to recover from prior pregnancies; giving up breastfeeding; the decreased financial impact on the family when births are spaced; time to care for the children they already have; and husbands also appreciating the break that contraceptives provide. Capacity to schedule a subsequent pregnancy based on factors such as work, finances, and support. Participants in the FGD reported that husbands occasionally supported the use of contemporary techniques. Particularly after the first one or two kids, this is true. As someone stated *“My husband asked me to insert the IUD. We require a rest.!” #10* Another participant echoed, *“My spouse provided me the money for the implant, and he made sure I used it.” #8* Another related: *“My husband gave me some cash and told me to go get the medication.” #7* Additionally, a few FGD participants mentioned favorable life outcomes as a result of utilizing contraceptives. Condoms are simple to use and preventing infection was one of them. A woman has a chance to relax between pregnancies, which is beneficial for her physically and psychologically. Implants, injectables, COCS, and IUDs are suitable for the body and simple to use. They can also assist to control menstruation and blood pressure. Contraceptive tablets can ease pain and control menstruation.

**Modern vs. traditional birth spacing methods:** participants in one FGD had the opinion that both traditional and modern methods produced the same results but that neither was completely effective and that there was still room for improvement in both areas. These individuals also stated that they thought contemporary procedures were more trustworthy than older ones. This was supported by participant comments: *“Women can forget to count, or irregular periods can make counting imprecise. The withdrawal approach is unreliable since husbands may object and they have no control over this, according to participants, who also made this statement...” #10* Traditional methods may be preferred in some situations for health-related reasons. Due to their dislike of having “foreign chemicals” in their body, two individuals preferred traditional treatments. This is consistent with what other participants have reported seeing when using injectable contraception. For three to four years after using injectables, several people who had used them reported being unable to become pregnant. These individuals have also heard that using injectables can cause periods to become irregular and that some women may go years without having a period. Reasons for refusing to use modern birth spacing methods participants in the FGD who were still using traditional techniques said that the major thing keeping them from switching to modern techniques was the effect it would have on their bodies and the potential for it to harm future fertility as said: *“Modern methods terrify me and all the adverse effects are concerning, and I think it's risky to put substances into your body.” #7* and *“These birth control methods are yet another means of eradicating us.” #2* Participants who were still using conventional procedures considered the IUD to be the worst type of modern approach because they had heard that IUDs cause more bleeding and pain. The pill attracted interest as well. Several individuals think that the pill is partly to blame for women feeling anxious, stressed, and gaining weight. However, the overall tenor of the FGDs suggested that participants would be open to switching from traditional to modern methods based on which techniques best fit their bodies and which ones were readily accessible and reasonably priced, with clear awareness messaging on myths and family planning.

### Family planning decision making

**Determining the number and timing of children:** in all FGDs, participants cited social and cultural pressures to have children early and the necessity of producing a male child. Participants had mixed views about deciding how many children they would like to have and when to have them. For some participants, discussions about the number of children were had with their fiancés or extended family before marriage. For other participants, the topic was not discussed at all: *“If a woman does not have a child in the first year, everyone thinks there is something wrong with her, and that she should go and see a doctor.” #6* Another participant related: *“I did not want another child so soon after the first baby, but I had so much pressure on me from the family. They kept saying to me “God is not giving you any more children”. #10* Another participant gave birth to her first kid, a daughter, while she was still enrolled in school. Although she was able to delay any additional pregnancies until she finished her education, she felt pressure to have a son because her first child was a girl. As a result, she became pregnant as soon as she finished her degree due of this pressure.

**People making decisions about family planning:** participants in the FGD reported that decisions about family planning and family size are influenced by extended family, particularly mothers-in-law. Comment from a participant: *“Because she wanted her to produce boys, her mother-in-law put pressure on her and influenced her husband...” #5* Another participant stated: *“Because I had only produced girls, my family and those around me kept telling me, “God willing, you will have about.”.” #9* After a first kid, some FGD participants said their husbands made the decision whether or not to have future children. The women also said that they talked to their husbands about their choice of contraception and that choosing a technique was frequently a collaborative decision. The size of participants' own families, the number of children their siblings had, and whether they had relatives who had previously been unable to conceive children all affected how much pressure and influence family members had on them. As an illustration, one participant said that: *“due to the fact that she was an only child and did not want this for her grandchild, she wanted her daughter to have children as soon as possible...” #4* Participants in the FGD did not agree on who ultimately decides whether to have more children. Occasionally, participants were given the authority to make this choice on their own; other times, couples made this choice together; and in several instances, participants reported that the husband was given the final say.

**Problems in decision-making in family planning:** participants in the FGD gave a variety of answers regarding the types of disagreements that exist in FP decision-making. Money-related difficulties are a recurring theme in FGDs. As one participant said: *“Because of the strain on our finances, my husband did not want us to have many kids. He initially stated we should stop at two children, though he would have preferred only one. He was devastated when I unintentionally became pregnant for a third time with twins. Yet I informed him that this was a gift from God.” #5* Other individuals claimed that they wanted to enjoy life while they were still young, which caused conflicts in the family. The current size of the household was another topic of contention. There were also differences of opinion on the usage of contraceptives. The majority of FGD participants concurred that birth spacing and the importance of family planning were subjects of conversation in their relationships. Participants in the FGD said that families assist in resolving conflicts and difficulties. Sometimes a couple will make a decision before consulting the rest of the family. Other individuals, on the other hand, said they consult their mothers, grandmothers, and sisters to determine the best course of action. Another person affirmed this and said *“If my husband and I can't agree on when to have the next baby, I'll talk to my family. It benefits both of us to have their experience...” #8* The need for the woman to take care of herself after giving birth, the need to avoid having the children too close together, the number of children, and the mother's health and readiness for another pregnancy were other issues that some participants mentioned coming to agreements with their spouses about.

### General family planning information

**Motives for selecting a specific family planning method:** participants in the FGD expressed a preference for IUDs, implants, and injectables. This was justified on the grounds that women may have it inserted and then forget about it. IUDs and implants don't contribute to weight gain either. Some participants also suggested the medication. Some participants supported old approaches and discouraged the use of contemporary ones. One participant disagreed with the notion of endorsing one technique over another. She remarked: *“I disagree with the notion of advising women to use contraceptives because I believe it depends on the woman's body and how she responds to it. Using an IUD might be extremely difficult for one woman while being completely painless for another. It fits the woman, after all.” #2*

**Access to information about family planning methods:** the FGDs revealed varying opinions about how easy it was to get accurate information. Participants occasionally said they didn't feel they had enough access to reliable information. In other instances, participants claimed to have access to a good deal of information on family planning and the methods that are currently accessible, and they believed they understood how modern methods functioned. One woman stated: *“We need to learn more about the available techniques because it doesn't seem like we have a choice. Because they are trying to sell us a certain product, some doctors just tell us what we should use without giving us a choice. The best outcome for us is unimportant to them. Another major issue is that some doctors are horrible at placing IUDs and implants. The woman may have pain and bleeding as a result.” #4* Participants in the FGD said that they used the following channels to get information both formally and informally: Hospitals and healthcare facilities, media and the internet, other women, friends and family, peer groups, and health care worker training programs

**Additional information requested about family planning:** participants in the FGD reported that they are interested in learning more about the adverse effects of contemporary birth control techniques, particularly long-term ones, as well as how they might affect a future pregnancy. As an illustration, one participant said: *“The advertising for the implant, male condoms, pills, and IUDs emphasizes their benefits but does not discuss any negative effects.” #6* Another person expressed the opinion that adopting contemporary procedures, such as the pill or an IUD, increases the risk of developing cancer. A different participant said: *“I need to understand what this implies for my body even though I have had an IUD for ten years. What the long-term implications are, I don't know.” #1* Additionally, participants appear open to and engaged in study and self-awareness. According to one participant: *“I read everything, including the instructions for the IUD, implants, and injectables. On the internet, I also look for information. I don't trust the doctor to provide me with the information I require...” #2* The availability of information to newlyweds and young women regarding the dangers of utilizing the withdrawal strategy was seen by some participants as being of particular importance. Additionally, participants sought information and suggestions on how to help their husbands learn more about family planning, as well as information geared exclusively toward guys [8].

**Information sources:** participants sought out information from hospitals and health facilities, healthcare professionals being trained, their own family, friends, and relatives, as well as from the Internet and social media. The FGDs differed in their assessments of the accuracy of peer-reported data. Some participants said they would like to acquire information from qualified healthcare professionals rather than simply relying on advice from friends or family since it is more reliable. Participants in the FGD concurred that the following routes provided the greatest information delivery: seminars or clinic-based health education events, Brochures, healthcare professionals, conversations with a professional, Television shows, fact sheets, clinic posters, Internet and WhatsApp.

**Affordability of contraceptives:** the majority of FGD participants agreed that prescription prices at the pharmacy make modern contraception techniques accessible. During campaigns or at health facilities, some participants receive complimentary tablets from the Ministry of Health (MoH).

**Influence of COVID-19 on the usage of family planning:** participants in the FGD reported a variety of opinions about how COVID-19 has affected the usage of family planning. Some individuals disagreed with the notion that COVID-19 has no effect on access to family planning because they thought it had an effect on it. According to one of the participants, COVID-19's effects *“I was on Sayana press and when the time for the next injection came it was when there was a lot of skepticism concerning covid, I had to be using but condom with my husband, and I believe many other couples were facing the same challenges we were having” #8*. Some participants however believe there was no hindrance to accessing family planning. As related by one participant *“I did not believe that during the corona period I had access because I was not on family planning and from giving birth to my daughter when I came for vaccination of my child, I decided to take implants and with this on I don´t have to go always to the hospital” #1*.

## Discussion

The understanding and perceptions of unmet FP requirements in the Buea Health District are thoroughly explored in this qualitative study. This research produced conclusions regarding the supply-side and demand-side barriers and challenges associated with the use of FP measures, steps that can be taken to improve their use, and the role of couples in boosting FP coverage. It also revealed the knowledge and perceptions of rural residents regarding FP and its methods. This was divided into four main themes: family planning attitudes, justifications for switching from traditional to modern family planning, decision-making towards family planning, and accessibility of FP in light of the COVID-19 epidemic. Having children earlier in marriage was a sensible choice for both health reasons and social expectations and pressures. Although having a child as soon as is practical is the greatest option, there are some drawbacks, such as the mother's maturity and financial stability. With a three- to five-year gap between them, children should ideally be born within the first two years of marriage. However, some researches claim that FP use is influenced by racial and religious diversity. Family planning measures were utilized more frequently by wealthy ethnic groupings than by impoverished populations [9]. Despite the fact that family planning services are largely free for all Cameroonian citizens. In this study, people who had relocated from conflict zones were more aware of and more frequently used FP services than people from the semi-urban group living in the neighboring area [9]. To explore the disparities in knowledge and views about FP between the population categories, more study is needed in this area.

One could argue that this is a drawback of the present FP promotion efforts, which may not have taken into account the various requirements of persons from various racial and religious backgrounds [9,10]. A few individuals claimed that their sacred texts banned them from utilizing FP procedures because they believed that children were a gift from God and that using any artificial means to end a pregnancy or eliminate the possibility of life was wrong in their eyes [11]. This notion is deeply ingrained in some groups in Cameroon, according to earlier research [12,13]. The physical side effects on a woman's body are the most important problems with modern birth control methods, including condoms, pills (postinor-2), implants, injectables, and IUDs. Despite the problems people have with contraception, there are some advantages to using it. Both contemporary and conventional FP techniques have a chance of failing [14]. Women are reluctant to use FP techniques for a variety of reasons, including religious convictions, fear of side effects, having had negative health impacts after using hormonal contraceptives, and worry about future infertility [15]. Furthermore, despite some women's high enthusiasm in adopting FP measures, it is possible that obstacles related to language, culture, and discrimination, particularly by male counterparts, prevent some of them from doing so. Demand could be increased by using IEC resources to empower married couples to make decisions together and to increase knowledge [13,14]. Local cultural taboos in the Buea Health District limit open discussion of safer sex practices and sexual health, preventing young girls and boys from receiving sufficient knowledge and counseling on sexual and reproductive health and FP [9,15].

Family members help to resolve family planning (FP) problems and issues. Extended families, particularly mothers-in-law, have a significant impact on decisions about family size and planning. Participants are interested in learning about the long-term negative effects of existing birth control methods, as well as how contraceptives will affect a potential pregnancy. Men frequently asserted that they make all critical family decisions, including those pertaining to family health and contraception. Most of the time, men make the decision to use the FP measure alone, without consulting their partners. This may be one of the factors preventing women from choosing the FP method they ultimately follow. In addition, the fact that men's FP options are limited to condoms and permanent sterilization only serves to exacerbate the issue. Other studies in South Asia, where family planning measures are typically thought to be the duty of women, support these findings [16]. Similar to earlier research in India [16,17] and Nepal [18], the most prevalent sources of information about FP were health professionals, peers, and the media. Participants in this study appeared to hold the other gender responsible for using FP. This can indicate that there is a communication gap between the partners when it comes to FP decisions. More research is required to determine how to enhance communication between spouses.

The majority of married men and women said that the family's decision-makers are men. In more precise terms, the husband determines whether or not to allow their wife to take contraception [19]. However, those who were not married reported their willingness to make future decisions about using FP in concert with their partner [12,16]. The majority of the women in this survey appeared at ease letting their male partners choose the method of birth control. The prevalence of patriarchy in decision-making may help to explain this mindset [9,20,21]. Participants' opinions on whether the COVID-19 environment affects their ability to acquire family planning are conflicting. The unmet need for contraception could be made worse by supply restrictions (distance to a provider for receiving contraceptives, out-of-stock, few options, expensive methods, etc.). The Nepali health system faces comparable restrictions on all regular supplies. However, supply-side measures including expanding the number of healthcare institutions offering FP services, focusing on constant business hours, and maintaining a full supply of a wide range of FP techniques could significantly boost uptake and boost the prevalence of contraception [17,22].

## Conclusion

The focus groups showed that participants are intelligent and nuanced in their understanding of important issues that have a big influence on them. Traditional and contemporary family planning methods are widely understood, despite the pressure that cultural and societal norms around family planning issues and choices continue to exert on women. The participants expressed a significant desire to gain access to a greater range of general and specialized family planning services, as well as to learn more about the alternatives to traditional family planning methods and how various techniques affect their bodies. The COVID-19 background instilled apprehension, but women are quickly overcoming these worries to have access to family planning.

**Recommendations:** to find the best manner for family planning users to learn about family planning methods, an intervention study should be conducted; to what extent do religion and culture contribute to the unmet need for family planning and in what ways and to what extent do in-laws and in-law parents impact African households should be studied.

### What is known about this topic

- *Sociocultural factors affect the utilization of contemporary family planning techniques;*
- *Despite all the negative effects, traditional ways were still used;*
- *The use of family planning techniques continues to be important in partner decisions*.

### What this study adds

- *The study raised awareness about the usage of contemporary birth control and family planning techniques;*
- *The results of this study will enhance our understanding of the variables influencing unmet family planning needs and help us design effective intervention strategies*.
